# Sequence Variation Analysis of HPV-18 Isolates in Southwest China

**DOI:** 10.1371/journal.pone.0056614

**Published:** 2013-02-25

**Authors:** Mengjie Shen, Xianping Ding, Tianjun Li, Gangyi Chen, Xiao Zhou

**Affiliations:** 1 Bio-resource Research and Utilization Joint Key Laboratory of Sichuan and Chongqing, Institute of Medical Genetics, College of Life Science, Sichuan University, Chengdu, People’s Republic of China; 2 Institute of Medical Genetics, College of Life Science, Sichuan University, Chengdu, People’s Republic of China; University of Regensburg, Germany

## Abstract

Intratypic variations of HPV-18 are known to differ in the persistence of the infection, frequency of carcinogenesis and the progression of precursor lesions to advanced cervical cancer. This study was designed to analyze sequence variations of HPV-18 isolates in order to discover novel HPV-18 variants and to evaluate the variations among infected women in southwest China. Cervical biopsies from 56 HPV-18-positive women with cervical neoplasia were assayed by PCR amplification and sequencing of all eight genes (E1, E2, E4, E5, E6, E7, L1, L2) of the HPV-18 genome. The most frequently observed variation was a C to G transversion at nucleotide 287 of E6, which was found in 48.2% of samples. Analysis of E7 revealed only one specimen as having sequence variations. In addition, we have identified several novel variations: A551C in E6, G6906A in L1, and C4915T and C5147A in L2. The mutations in E6 and L2 are silent, while the E7 mutation results in a single amino acid change. This study complements and expands on previous descriptions of HPV-18 variants. The sequence variation data presented here provides a foundation for future research on HPV-induced oncogenesis and may prove valuable for developing diagnostic probes and in the design of HPV vaccines for targeted populations.

## Introduction

Cervical cancer is the second leading cause of cancer-related deaths in women worldwide [1]. Every year, more than 500,000 new cases of cervical cancer and roughly 250,000 deaths are recorded [2]. Nearly all cervical cancers are caused by human papillomavirus (HPV) infection [1].

HPV is a double-stranded circular DNA virus with a genome size of about 8000 bp that encodes early proteins (E1, E2, E5, E6 and E7) and late proteins (L1, L2 and E4) [3], [**4**], [**5**]. As described by de Villiers et al. [6], the sequence of the highly conserved L1 ORF is used to classify HPV isolates. Isolates with a L1 sequence more than 10% different than the nearest HPV type are a distinct “type”, while isolates with differences of less than 2% are termed “variants.” There are 120 different HPV types identified to date [6], [7], of which 15 have been termed “high-risk” types due to their significant association with cervical cancers [8]. There are 25 HPV types with strong, sufficient, or limited evidence of causing cervical cancer [9]. The types were classified and assessed by the IARC Monograph Working Group. Among them, the HPV-16 was the most oncogenic HPV type, known to cause cancer at 7 sites. HPV-18,31,33,35,45,52,58 were frequently found in cancer, while HPV-39,51,56,59 appeared less frequently [10]. Taking everything into account, HPV-16 and HPV-18 represent the most commonly identified high-risk HPV genotypes, which cause 40–60% and 10–20%, respectively, of all cervical cancers [11], [12].

In addition to the diversity among types of HPV, there are many natural intratypic variants, some of which contain alterations that lead to changes in the amino acid (AA) residues of functional and/or antigenic domains. Some of these changes have been shown to influence the persistence of the infection, morbidity of carcinogenesis, and the progression of precursor lesions to cancer [13], [**14]**, [**15**]. At present, analyses of sequence variations of HPV-16, which occur in early genes, late genes, and the upstream regulatory region (URR or long control region, LCR), have been relatively comprehensive [16], [17], [18], [**19**]. In contrast, data on HPV-18 variants is limited, and sequence variations of HPV-18 in the Asian population have been evaluated even less than those in European and American populations. The present study examined a collection of 56 different isolates of HPV-18 from cervical cancer patients in the southwest China and analyzed the sequence variations in the E1, E2, E4, E5, E6, E7, L1 and L2 viral genes. The data presented here is useful for future research on viral persistence, transmission and oncogenic potential, and may provide critical information for developing diagnostic probes and as well as designing vaccines for a specific population.

## Materials and Methods

### 2.1 Ethics Statement

The study was approved by the Education and Research Committee and the Ethics Committee of Sichuan University (approval # SCU20100056494). Written informed consent was obtained from each patient, who granted us permission to use the data obtained in subsequent studies.

### 2.2 Specimens

Specimens were obtained from the cervical biopsies of 56 women with cervical cancer who tested positive for HPV-18 at the Cancer Hospital of Sichuan Province and the 4^th^ People’s Hospital of Chongqing. Medical background information was recorded from all patients.

### 2.3 Genomic DNA Extraction

DNA was extracted using a commercial kit (U-gene DNA kit) according to manufacturer’s instructions (AnHui U-gene Biotechnology Co., Ltd. AnHui 242000, PR. China).

DNA was extracted and stored in a designated area free from potential DNA contaminants.

### 2.4 PCR

PCR reagents were mixed under a biosafety-hood in a designated room that was free from potential DNA contaminants. Amplification of the integrated HPV genes was performed with primers that were designed according to the HPV-18 reference sequence published in GenBank (accession number NC_001357) (Table 1).

**Table 1:** Primers used for PCR.

HPV-18 region	primer	Primer position	primer nucleotide sequence	Product size (bp)
E1	E1-1F	867	5′- CCCTGTCCTTTGTGTGTCCGT-3′	612
	E1-1R	1478	5′-TATTGCTATTGTCACTTGTACCGTC-3′	
	E1-2F	1436	5′- GGCAACAACAGCAGTGTAGACGGTA-3′	661
	E1-2R	2096	5′-TGCTGTTGCTGTCTGCTAATAAGGC-3′	
	E1-3F	2038	5′- GCTGACAGATGAAAGCGATATG -3′	433
	E1-3R	2470	5′-CGGTTCCAACCAAAAATGACTAGTG-3′	
	E1-4F	2361	5′- GTGGACCAGCAAATACAGGAAAATC -3′	561
	E1-4R	2921	5′-CTGTATTTGGCTGTCTATGTC-3′	
E2	E2-1F	2814	5′-AGATGCAGACACCGAAGGAAAC-3′	632
	E2-1R	3445	5′-TCGTCACTGGTACTGCACATAGA-3′	
	E2-2F	3420	5′- GACTCTATGTGCAGTACCAGTGACG-3′	599
	E2-2R	4018	5′-ATACAGACAGATGGCAAAAGCGGGA-3	
E4	E4F	3296	5′-GGGATTGTATTATGTAAAGGAAGGG-3′	456
	E4R	3751	5′-TGGTCGCTATGTTTTCGCAATCTGT-3′	
E5	E5F	3885	5′- CAAATATTGGTGGGATACATGAC-3′	380
	E5R	4264	5′- TGCGGCACGGTGGGATACCATACTT-3′	
E6	E6F	85	5′- AGAAACACACCACAATACTATGGCG -3′	662
	E6R	746	5′-GTCGGGCTGGTAAATGTTGAT-3′	
E7	E7F	540	5′- CGACAGGAACGACTCCAACGA-3′	431
	E7R	970	5′- ATAAAACCAGCCGTTACAACCCGTG-3′	
L1^a^	L1-1F	5402	5′- GTAACGGTCCCTTTAACCTCCTC-3′	650
	L1-1R	6051	5′- 5′- CATTGTCCCTAACGTCCTCAG-3′	
	L1-2F	6002	5′- AAGTTCCCATGCCGCCACGTCTAAT-3′	505
	L1-2R	6506	5′- AGAGCCACTTGGAGAGGGAGAATAC-3′	
	L1-3F	6502	5′- GCTCTATTGTTACCTCTGACTCC-3′	636
	L1-3R	7137	5′- ATTACTTCCTGGCACGTACACGCAC-3′	
L2	L2-1F	4239	5′- AAAGTATGGTATCCCACCGTGCCGC-3′	518
	L2-1R	4756	5′-TGGAACTTCAATAATGGACGGA-3′	
	L2-2F	4755	5′- CACAAACTGGGGAGGTGGCAGGTAA-3′	527
	L2-2R	5281	5′-GTCATTGTCCTCCGTGGCAGATACT-3′	
	L2-3F	5259	5′- TATCTGCCACGGAGGACAATGACTT-3′	398
	L2-3R	5656	5′-CTGTTATGGCATATAGAAGGTGG-3′	

Because of the gene is too long, E1, E2, L1 and L2 were divided into some subunits to design primers for future sequencing and variation analysis. F, upstream primer; R, downstream primer.

The PCR amplification was performed under the following conditions. An initial 5 min denaturation step at 95°C was followed by 35 amplification cycles, with each cycle including a 45 s denaturation step at 94°C, a 45 s annealing step at 55°C, and a 60 s elongation step at 72°C. Amplification was complete after a final 10 min elongation step at 72°C. Each 50 µl PCR reaction contained 4 mmol/L MgCl_2_, 400 µmol/L dNTPs, 3 U Taq DNA polymerase and 4 pmol primers.

Amplicons were visualized on 1.0% agarose gels stained with ethidium bromide under UV transillumination (data not shown).

### 2.5 Sequencing and Variations Analysis

PCR products were automatically sequenced using the Big Dye Terminator v3.1 Cycle Sequencing Kit (Applied Biosystems) according to the manufacturer’s instructions. The sequences were subsequently analyzed by NCBI Blast and DNAMAN version 5.2.2. HPV-18 DNA nucleotide positions were numbered according to the HPV-18 reference sequence (NC_001357).

All data were confirmed by repeating PCR amplification and sequence analysis at least twice.

## Results

In this study, nucleotide (nt) sequences of E1 (nt 914 to 2887), E2 (nt 2817 to 3914), E4 (nt 3418 to 3684), E5 (nt 3936 to 4157), E6 (nt 105 to 581), E7 (nt 590 to 907), L1 (nt 5430 to 7136) and L2 (nt 4244 to 5632) were determined. Sequence variations were identified in 33 for the 56 specimens assayed (Table 2). In 23 of 56 (41.1%) specimens, the nucleotide sequences corresponded exactly to the HPV-18 reference sequence (NC_001357).

**Table 2:** Sequence variations of HPV-18.

regions	E6		E7		E1	E2			E2			L2		L1					
Nucleotide position	287	551	640	864	2856	2857	2858	2859	3084	3085	3275	4915	5147	5503	5701	6460	6625	6842	6906
reference	C	A	C	A	T	G	C	G	C	G	C	C	C	G	C	C	C	C	G
variation	G	C	T	G	G	C	G	T	G	C	G	T	A	A	G	G	G	G	A
Amino acid sequence	P	R	P	N92S	L648C	C14A	R649V	V15L	R 9 0 A		T	P	R	R25Q	P91R	P344R	P399R	P	D493N
cc-03	G													A	G	G	G	G	
cc-05	G													A	G	G	G	G	A
cc-06	G													A	G				A
cc-07	G				G	C	G	T	G	C		T	A	A	G	G	G	G	
cc-10	G	C			G	C	G	T	G	C	G		A			G	G	G	A
cc-12												T		A	G	G	G	G	
cc-15	G													A	G	G	G		A
cc-16					G	C	G	T				T	A				G	G	
cc-18	G	C			G	C	G	T	G	C	G	T	A			G			
cc-19					G	C	G	T						A	G	G	G	G	
cc-20					G	C	G	T	G	C									
cc-21	G													A	G	G	G	G	A
cc-22	G								G	C	G		-	A	G	G	G	G	
cc-25	G		T	G										A	G		G	G	
cc-26	G	C			G	C	G	T	G	C	G	T		A	G	G			
cc-27	G				G	C	G	T	G	C	G	T		A	G	G			
cc-29	G	C			G	C	G	T					A				G	G	
cc-30	G													A	G	G	G	G	A
cc-33					G	C	G	T				T	A			G	G		
cc-35	G	C												A	G				
cc-36	G	C			G	C	G	T	G	C	G	T	A	A	G		G	G	A
cc-37					G	C	G	T	G	C	G			A	G	G	G	G	A
cc-39	G								G	C						G			
cc-43	G													A	G	G	G	G	
cc-44	G				G	C	G	T				T	A				G	G	
cc-45	G				G	C	G	T	G	C				A	G	G	G	G	A
cc-46	G				G	C	G	T											
cc-47	G													A	G	G	G	G	
cc-48	G	C			G	C	G	T							G	G	G	G	A
cc-49	G	C												A	G	G	G	G	A
cc-52	G											T	A	A		G	G	G	
cc-53	G				G	C	G	T				T	A	A	G	G	G	G	A
cc-56	G	C			G	C	G	T						A	G	G	G	G	

Sequence variations of HPV-18 E1, E2, E6, E7, L1 and L2 were based on the HPV-18 reference sequence (NC_001357). The identification codes of the specimens are indicated along the far left column. The number and the letter represent the nucleotide variation at that position. Blanks indicate no variation. In the ‘reference’ row, the letters represent the nucleotides in the reference sequence. In the ‘amino acid sequence’ row, the number and the letter represent the AA change at that position.

### 3.1 HPV-18 E1 Sequence Variations

**Figure 1:**
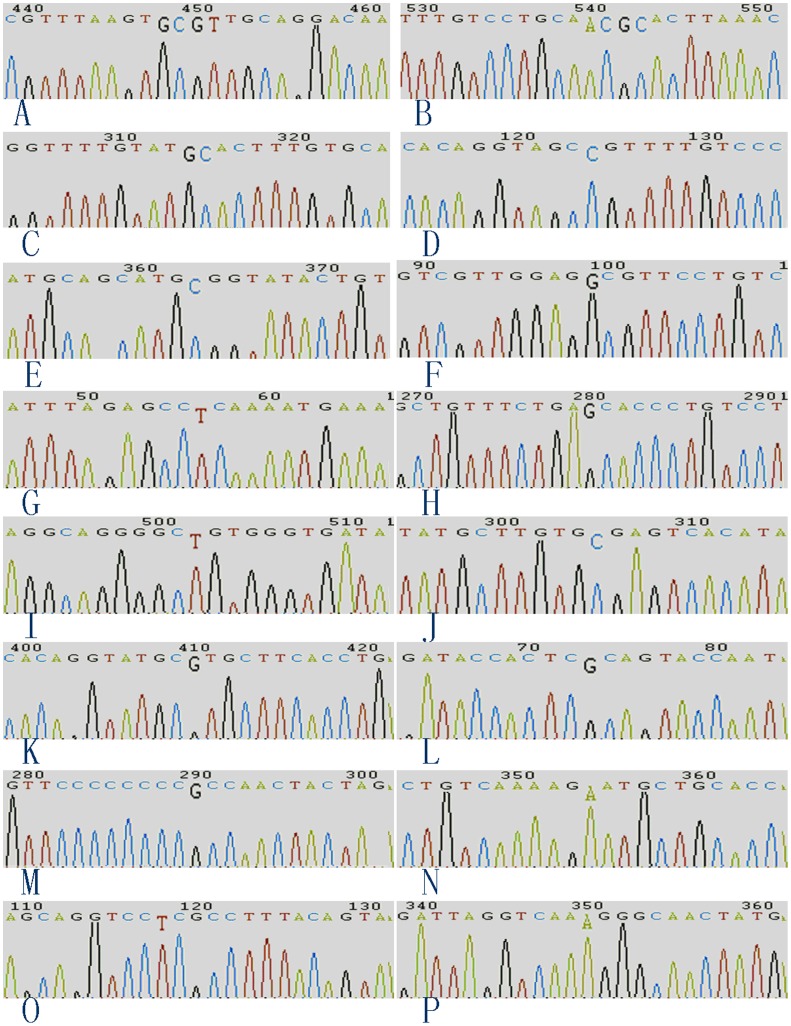
Chromatograms of sequence variations. The sequence variations were enlarged to facilitate observation. In B, D, E, F, I and J, the reverse complement of the sequence variations are displayed because the sequences were obtained using downstream primers. G, guanine; C, cytosine; A, adenine; and T, thymine.

As shown in Fig. 1A, DNA sequence analysis of the HPV-18 E1 region revealed the following four variations: a T to G transversion at nt2856 and a G to C transversion at nt2857 leading to a L648C AA substitution (n = 18, 32.1%), and a C to G transversion at nt2858 and a G to T transversion at nt2859 leading to a R649V AA change (n = 18, 32.1%). The four variations were shared in E1 and E2.

### 3.2 HPV-18 E2 Sequence Variations

DNA sequence analysis of the HPV-18 E2 region revealed seven variations: a T to G transversion at nt2856, G to C transversion at nt2857 and C to G transversion at nt2858, leading to a C14A AA substitution (n = 18, 32.1%); a G to T transversion at nt2859, leading to a V15L AA substitution (n = 18, 32.1%) (Fig. 1B); a C to G transversion at nt3084 and a G to C transversion at nt3085, leading to a R90A AA change (n = 11, 19.6%) (Fig. 1C); and a C to G transversion at nt3275, which does not lead to an AA change (n = 7, 12.5%) (Fig. 1D). Among these variations, T2856C, G2857C, C2858G and G2859T were simultaneous, as were C3084G and G3085C.

### 3.3 HPV-18 E6 Sequence Variations

DNA sequence analysis of the HPV-18 E6 region revealed two variations that did not lead to AA changes. The most frequent variation was a C to G transversion at nt287 (n = 27, 48.2%) (Fig. 1E), with a less frequent A to C transversion occurring at nt551 (n = 9, 16.1%) (Fig.1 F).

### 3.4 HPV-18 E7 Sequence Variations

DNA sequence analysis of the HPV-18 E7 region revealed two variations, both of which occurred only once and in the same specimen: a C to T transition at nt640, which did not result in an AA change (Fig. 1G), and an A to G transition at nt864, leading to a N92S AA substitution (n = 1, 1.79%) (Fig. 1H).

### 3.5 HPV-18 L1 Sequence Variations

DNA sequence analysis of the HPV-18 L1 region revealed six variations: a G to A transition at nt5503, leading to a R25Q AA substitution (n = 23, 41.1%) (Fig. 1I); a C to G transversion at nt5701 with an AA change of P91R (n = 23, 41.1%) (Fig. 1J); a C to G transversion at nt6460 with an AA change of P344R (n = 24, 42.9%) (Fig. 1K); a C to G transversion at nt6625 with an AA change of P399R (n = 25, 44.6%) (Fig. 1L); a C to G transversion at nt6842 with an AA change of P471R (n = 23, 41.1%) (Fig. 1M); and G to A transition at nt6906 with an AA change of D493N (n = 12, 21.4%) (Fig. 1N).

### 3.6 HPV-18 L2 Sequence Variations

DNA sequence analysis of HPV-18 L2 region revealed two variations that did not lead to AA changes: a C to T transition at nt4915 (n = 11, 19.6%) (Fig. 1O) and a C to A transversion at nt5147 (n = 10, 17.9%) (Fig. 1P).

### 3.7 HPV-18 E4 and E5 Sequence Variations

No variations were detected in the HPV-18 E4 and E5 regions.

## Discussion

A study by Angulo et al. [20] highlighted the fact that co-infection with more than one HPV type is not a rare occurrence, and this situation can lead to HPV recombination and the generation of new HPV types. Therefore, it is of the utmost importance to gather information on HPV variants and analyze different genomic regions and cases of multiple infections for developing HPV diagnostics, vaccines and other therapeutic approaches to manage viral-induced cancer [16]. HPV-18 variants have been shown to have different biological as well as biochemical effects, as some variants can result in greater transforming efficiency, more aggressive clinical outcome, higher probability of cancer recurrence and worse prognosis [21], [22], [23], [24]. In this study, we described the HPV-18 intratypic diversity over a span of 7000 nucleotides among isolates from southwest China. Our data set on E1, E2, E4, E5, E6, E7, L1 and L2 sequence variations of HPV-18 complements and expands on previous descriptions of HPV-18 variants, which were based on a targeted analysis of E6, LCR-E6, E2, L1 and URR [23], [25], [26].

Among the early gene products, the E1 viral protein plays a critical role in controlling viral replication and load, and it requires the interaction with the E2 protein to bind to the LCR [27]. Whereas E1 viral helicase is the replication initiator protein, E2 is the major viral regulator of viral transcription and replication, repressing transcription of the E6/E7 oncogenes, and together with the E1 helicase, activating viral DNA replication [28].

The E2 protein contains three distinct domains: a transactivation domain (TAD), an unstructured hinge domain, and a DNA binding domain (DBD) [28], [29]. We discovered that all of the E2 sequence variations in our set of samples are located in the TAD (2856–2859) and the unstructured hinge domain (3084, 3085 and 3275). None were found in the DBD domain, which is not unexpected given that the sequence of the DBD is highly conserved due to its role in binding a specific consensus sequence (ACCN_6_GGT) [28], [30], [31] in the LCR of the viral genome.

Mutagenesis analysis of the E2 TAD indicated that most of the conserved AAs in this domain are required for both transcriptional activation and viral DNA replication [25], [32]. Moreover, Cooper et al. [33] proposed that the N-terminus of E2 is tightly configured, as single AA substitutions have been found to impair its ability to support replication. Our study identified four variations in the TAD domain at the N-terminus. These changes resulted in two AA changes: C14A and V15L. Both the C14A and V15L amino acid substitutions occur in the first amino-terminal α-helix of the TAD domain [34]. In a study by Harris and Botchan, both structural and mutagenesis data indicate that this region is more important for HPV transcription than replication [35]. To the best of our knowledge, no functional assignment of these residues has been made at this time.

E1 shares the sequence variations at nt2856–2859 with E2 in TAD, through which E2 interacts with the E1 helicase [5]. These variations result in L648C and R649V AA substitutions in the E1 coding region, especially, in the C-terminal helicase/ATPase region, which unwinds the HPV genome to allow for replication [36]. The importance of these residues to the ATP-dependent DNA helicase activity is unknown at this time, and elucidating functional changes brought about by these substitutions is an important area of future investigation.

E6 and E7 encode the main viral proteins involved in human epithelial cell immortalization and transformation. The oncoproteins are able to increase the expression of genes related to DNA synthesis and coordinate cell cycle induction, thereby increasing the productive life cycle of HPV [37]. The E6 protein induces cell proliferation by interacting with numerous cellular proteins that are involved with tumor suppression, including p53 [38], [39]. In our data set, the most frequent variation was the C to G silent mutation at nt287. Furthermore, the guanine at E6 coding region nt183 is represented as HPV-18 prototype (AY262282). These results are in good agreement with a study by Chang et al. [26] in Taiwan, who reported the identical nucleotide variation, and suggests that the C to G variation at nt287 may be a common mutation worldwide. Since this frequent variant is a silent mutation, it may be used as a biomarker to track and study viral transmission [26].

The E7 protein interacts with another cellular tumor suppressor, pRb, and disrupts its normal function in regulating the cell cycle, thereby stimulating expression of many E2F-regulated transcripts [38]. E7 encodes a protein of about 100 AAs and contains three conserved regions: CD1, CD2 and CD3 [38]. Our sequencing results showed that HPV-18 E7 is highly conserved, with only one specimen detected as having sequence variations at nt640 and nt864; these variations were also detected in all African variants by Arroyo et al. [40]. The transition C to T at nt640 is a silent mutation, and A to G transition at nt864, leading to a N92S AA substitution. The N92S AA substitution is in the region linking CD2 and CD3. There is an optimal zinc-sensitive tertiary structure of HPV-18 E7, which modulates its interaction with target cellular proteins [41]. Indeed, the E7 domain of HPV-16 and HPV-18 is highly conserved [15], [16], [**17]**, [**38**], which strongly implies that it encodes the major transforming function [42], [43], and that the conserved domains are critical for the transforming activities of each of these viral oncoproteins [44], [**45**]. A recent study by Todorovic and colleagues suggests that the N92S substitution may have functional significance [46]. This residue was predicted to be exposed on the surface of the CR3 domain of E7 protein, and its mutation in HPV-16 (P92A) led to reduced dimerization of E7 (according to yeast two-hybrid analysis) as well as increased transformation potential. Thus, it is possible that this variant increases the incidence and severity of cervical cancer in infected women.

Changes in the L1/L2 regions of the HPV genome may be important in determining the infectious potential of different variants, as well as in defining epitopes relevant to vaccine design [47], [48]. The L1 protein is the main component of the viral icosahedron capsid and is capable of self-assembly, thus giving rise to virus-like particles [49]. The analysis of the L1 gene is of immense importance because of its high diagnostic value [16], as the range of intratypic variations observed in this region allows the distinction and assessment of known or novel HPV types [7], [50].

In the L1 coding region, one A to G transition at nt5503 and four C to G transversions were found. These five variations were also observed in a study by Arias-Pulido et al. [25] in the United States. Furthermore, the four guanine variations aare represented in the HPV-18 prototype sequence (AY262282), indicating that these variations are wide-spread. In addition, we also found a novel G to A transition at nt6906. All five sequence variations near C-terminus described above result in AA changes which may affect immune responses to HPV-18 capsid protein [25], [47]. The HPV L1 model postulates that the L1 C-terminal domain is exposed on the viral surface and expected to be highly antigenic [51].

Like L1, the L2 protein plays an important role in the initial steps of papillomavirus infection [52]. We found two sequence variations in the L2 ORF that did not result in AA changes. These changes could reflect the extreme rarity of the reference sequence that was originally obtained from a subject in southwest China.

Interestingly, no sequence variation was found in E4 and E5, in contrast to a report by Arias-Pulido et al. [25] in the United States, in which there were seven sequence variations found in E4. This discrepancy may be attributed to differences in sample size as well as the geographic and racial characteristics of the study populations.

### Conclusions

In summary, this study reports sequence variations in the E1, E2, E6, E7, L1 and L2 genes of HPV-18 isolates from southwest China. The sequence variations in the genes examined may contribute to HPV oncogenesis. Our data will provide a solid foundation for further biological and clinical studies, and may also be employed in epidemiological studies where sequence variations are used as markers for monitoring HPV infections in target populations. Elucidating the functional and pathological differences between intratypic variants of HPV, as well as determining the molecular basis for their propensity to induce different histological types of cervical cancer, are important areas of future investigation. Further studies of HPV sequence variation will also need to include different geographic regions with distinct and isolated human populations.
